# Optimization of Tensile Strength and Young’s Modulus of CNT–CF/Epoxy Composites Using Response Surface Methodology (RSM)

**DOI:** 10.3390/ma15196746

**Published:** 2022-09-28

**Authors:** Md. Rezaur Rahman, Nur-Azzah Afifah Binti Taib, Mohammed Mahbubul Matin, Mohammed Muzibur Rahman, Muhammad Khusairy Bin Bakri, Taranenko Pavel Alexanrovich, Sinitsin Vladimir Vladimirovich, Khairuddin Sanaullah, Diana Tazeddinova, Afrasyab Khan

**Affiliations:** 1 Faculty of Engineering, Universiti Malaysia Sarawak, Jalan Datuk Mohammad Musa, Kota Samarahan 94300, Sarawak, Malaysia; 2 Department of Chemistry, University of Chittagong, Chittagong 4331, Bangladesh; 3 Department of Chemistry, Center of Excellence for Advanced Materials Research (CEAMR), Faculty of Science, King Abdulaziz University, P.O. Box 80203, Jeddah 21589, Saudi Arabia; 4 Composite Materials and Engineering Center, Washington State University, 2001 East Grimes Way, Pullman, WA 99143, USA; 5 Research Institute of Mechanical Engineering, South Ural State University, 454080 Chelyabinsk, Russia; 6 Research Laboratory of Technical Self-Diagnostics and Self-Control of Devices and Systems, South Ural State University, 454080 Chelyabinsk, Russia; 7 Department of Food Technology and Organization of Public Catering, South Ural State University, 454080 Chelyabinsk, Russia

**Keywords:** electrophoretic deposition (EPD), volume ratio, deposition voltage, deposition time, CNT–CF/epoxy composite, response surface methodology, ANOVA, tensile strength, Young’s modulus

## Abstract

Composites such as carbon fiber are used extensively by automotive, aerospace, marine, and energy industries due to their strong mechanical properties. However, there are still many areas it is lacking in testing, especially related to its electrophoretic deposition. In this research work, the tensile strength and Young’s modulus of CNT–CF/epoxy composites were measured using the tensile test by varying the electrophoretic deposition (EPD) process parameters. Response surface methodology (RSM) was used to optimize the three main parameters in this EPD process: the volume ratio (water as the basis), deposition voltage, and time to obtain the maximum tensile properties of the composites. There were four volume ratios (0%, 20%, 80% and 100%) used in this design of experiment (DoE) with ratios’ pairs of 0%, 100%, and 20%, 80%. For this study, water and methanol were used as the suspension medium. This design’s deposition voltage and time were 10 to 20 V and 5 to 15 min. ANOVA further verified the responses’ adequacy. The optimum conditions for the first Design of Experiment (DoE) (0% and 100%) were identified as a volume ratio of 99.99% water, deposition voltage of 10 V, and 12.14 min. These conditions provided the maximum strength of these composites with a tensile strength of 7.41 N/mm^2^ and Young’s modulus of 279.9 N/mm^2^. Subsequently, for the second DoE (20% and 80%), tensile strength of 7.28 N/mm^2^ and Young’s modulus of 274.1 N/mm^2^ were achieved with the ideal conditions: volume ratio of 44.80% water, deposition voltage of 10.04 V, and time of 6.89 min. It can be concluded that the ideal interaction between these three EPD parameters was necessary to achieve composites with good tensile properties.

## 1. Introduction

Carbon fiber (CF)/epoxy composites, also known as carbon fiber-reinforced polymer composites (CFRPs), are used extensively in various automotive, aerospace, marine, and energy replacements of conventional metal materials. High strength and stiffness combined with moderately low density have increased the industry demand for CFRPs [1]. CF_S_ are used extensively in lightweight polymer composite materials as reinforcement materials owing to their excellent properties, such as electrically conductivity and low coefficient of thermal expansion, while still being chemically inert [2]. Moreover, epoxy has long-term dimensional stability, good adhesiveness, and excellent chemical, thermal, and mechanical resistance. These excellent properties made this thermoset polymer widely used as a matrix in fiber-reinforced polymer (FRP) composites [3]. The interfacial interactions of the CFs and epoxy matrix greatly influence the performance of CFRPs, especially their mechanical properties [4]. The load transfer from the “weak” matrix to “strong” fiber and from fiber to thread through the matrix is guaranteed by the interphase, known as the third phase. The interphase has noticeable physicochemical properties. The fiber/matrix adhesion influences the interphase’s load transfer capability [5]. However, interactions between the fiber and matrix for the CF and resin epoxy are not favored, as the CF surface is non-polar, while the epoxy resins are polar [6]. Additionally, CF surfaces that are chemically inert, hydrophobic, and intrinsically smooth have caused low interfacial bonding strength between the CF and the polymer matrix [7].

Hence, to overcome the inertness of the CF and obtain strong fiber/matrix interfacial adhesion, modifications should be done at the fiber surface [8]. The composite properties would be better when the fiber/matrix interfacial interaction is stronger. Hence, many studies have been done to enhance the bond strength, including integrating different types of nanoparticles onto the fiber’s surface [9]. Studies have shown that the CFRPs’ interfacial properties could be improved by introducing carbon nanotubes (CNTs) onto the reinforcing CF [10]. Owing to their outstanding mechanical properties, the addition of CNTs may effectively enhance the CF–matrix interaction [11]. In addition, the CFs’ surface roughness is improved with the presence of CNT and subsequently increases the CF–matrix interfacial adhesion [12]. Not only taking advantage of the excellent properties of CNTs, but the reinforced FRP material also maintained the superiority of the conventional fiber reinforcements. Strength, crackles, wear resistance, and rigidity is the properties in the original materials which could be enhanced with CNTs as reinforcement [13].

CNT–CF hybrids can be fabricated using several methods such as chemical vapor deposition (CVD), spray coating, dip coating, electrophoretic deposition (EPD), etc. Then, the nanoparticles are successfully deposited or attached to the CF surface. EPD has several advantages among these methods and is thus used for CF surface modification. Good surface homogeneity, control over film deposition, high deposition rate, and ease in process up-scaling make EPD the more favorable method [7]. Thus, EPD was chosen as the deposition method for the CNT onto the CF surface in this study.

Electrophoretic deposition (EPD) is a colloidal process in which electric fields are introduced in a suspension medium. The particles compactly packed in the medium are impelled onto the substrate [14,15]. Therefore, it is essential to use stable suspension media throughout the EPD process to ensure that the particles are dispersed stably [16]. Water is commonly used in the EPD process for several reasons: cost-effectiveness, low requirement of an electric field, ease of temperature regulation throughout the process, and environmentally compatible [17]. However, water electrolysis may occur when high voltage is applied, compromising the quality of the depositions. Organic solvents such as alcohols are used to overcome this issue. However, a high applied voltage is needed for pure organic solvents. Their particle mobility is also low due to the small electric charge on the particles. This can be overcome by combining organic solvent and water as the solvent for the EPD process [18]. For this study, methanol (CH_3_OH) was the chosen organic solvent.

There are two groups of parameters that characterize the EPD process, i.e., parameters related to the suspension stability, and those related to the deposition process. Six parameters are associated with the strength of the suspension, namely, particle size, the dielectric constant of liquid, the conductivity of suspension, suspension viscosity, the zeta potential, and suspension stability. Moreover, the deposition process is influenced by four parameters: deposition time, solid concentration in suspension, applied voltage, and substrate conductivity [19].

For this study, the effect of the parameters, suspension medium, deposition time, and voltage was investigated during the EPD process on the tensile properties of the composites. The interaction of those parameters was observed and optimized using response surface methodology (RSM). RSM combines the mathematical and statistical techniques to construct models by evaluating the effect of multiple independent variables to achieve the variables’ optimum values, obtaining desirable outcomes [20]. The EPD process was used to develop the experimental model using RSM to obtain the optimum input and output factor values. The effect of the CNTs on the fiber composites was ascertained by evaluating the composites’ tensile properties, scanning electron microscopy (SEM), and Fourier transform infrared (FTIR) spectroscopy. Furthermore, optimization was carried out to determine the ideal value of parameters from the design data, which exhibited maximum mechanical properties of the composites by RSM using Design Expert (StatEast Minneapolis, MN, USA) software (version 11).

## 2. Materials and Methods

### 2.1. Materials

The solution used for the EPD process consisted of two solvents, namely, methanol, and water. The methanol (CH_3_OH) was supplied by Merck kGaA (Darmstadt, Germany), whereas water was obtained using an Arium Pro UV Ultrapure water system (Sartorius, Göttingen, Germany). The MWCNTs (Product No. 0552CA) were from SkySpring Nanomaterials, Inc. (Houston, TX, USA). These MWCNTs were 95% purity, <8 nm in size, and in black powder form.

The carbon fibers used in the experiment were black plain weave CF supplied from PiCarbon (Kuala Lumpur, Malaysia). In addition, Epocast PT100 (clear type epoxy resin) and Epoharden PT100S (amine-based epoxy hardener) were obtained from Portal Trading (P.T.) (Pulau Pinang, Malaysia) and were used as matrix and curing agents, respectively.

### 2.2. Methods

#### 2.2.1. Design of Experiments

The effects of various combinations of input factors, i.e., volume (% *w*/*w*), voltage (V), and time (min), on the tensile properties of the hybrid CNT–CF/epoxy composites were investigated using a central composite design (CCD) response surface study. The values of the input factors are indicated in Table 1 and Table 2**,** where 2 pairs of different volume ratios were used in the experiment. The design generated 18 runs of varying factor conditions for each volume ratio. The tabulated experimental designs are shown in Table 3 and Table 4.

#### 2.2.2. Fabrication of the Hybrid MWCNT–CF Strips

**Figure 1:**
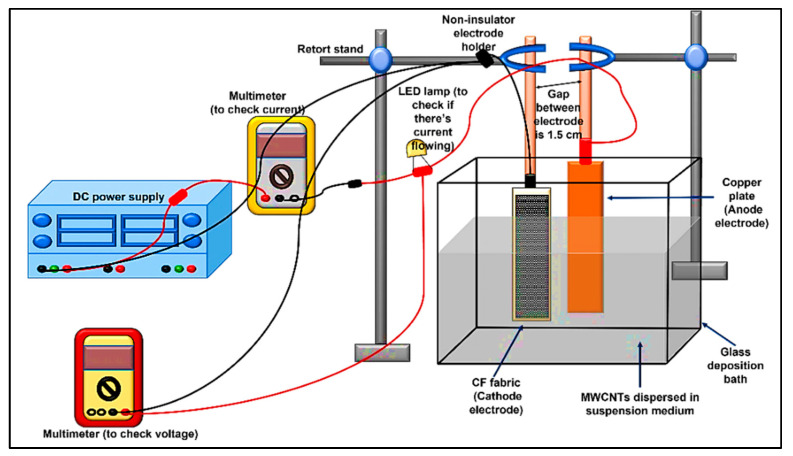
Schematic diagram of the EPD process for the fabrication of hybrid MWCNT–CF materials.

Before the EPD process, the suspension mediums were prepared by placing 80 mL of methanol (MeOH), water (W), and the mixtures of MeOH/W (as per experimental design) in laboratory glass sample bottles. The MWCNTs were dispersed in each suspension medium with the MWCNTs suspension of 0.05 mg/mL (0.004 g of MWCNTs were dispersed in 80 mL of solvent). The suspension was then ultrasonicated using an ultrasonic cleaner (sonicator) (KUDOS SK2200HP, Shanghai, China) for 15 min to ensure uniform dispersion of the MWCNTs before pouring it into the glass deposition bath. The EPD was performed following the above obtained experimental design. The experimental set-up of the EPD process consisted of a direct current (DC) power supply (Model 1672, B&K Precision, CA, USA), 2 multimeters (Fluke 115, Fluke, USA, and UNI-T UT61C, Batronix, Germany), copperplate electrode, mini-LED, and glass deposition bath, as illustrated in Figure 1. Alligator clips attached the CF and copper plate to the non-insulator electrode holder. The gap between the two electrodes was fixed at 1.5 cm using a retort stand, and the electrodes were then immersed in the MWCNTs suspension. This study used the CF as the cathode, while the copper plate was positioned as the anode (counter electrode). The EPD process was then carried out as per the design of the experiment, as shown in Table 3 and Table 4.

The average size of the deposited MWCF–CNT strip via the EPD process was 7.7 cm × 1.8 cm. After the EPD process, the deposited CF strip was dried at 70 °C for 12 h [21].

#### 2.2.3. Composite Preparation

**Figure 2:**
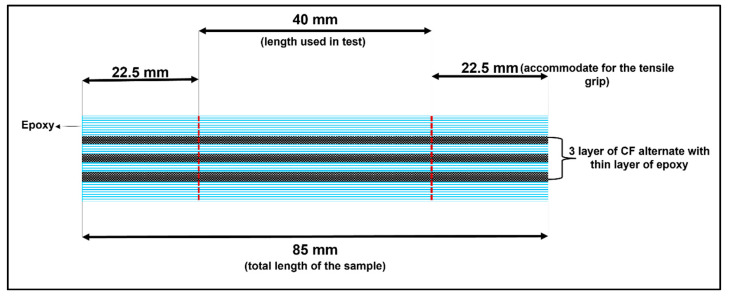
Schematic illustration of composites and their dimension.

The matrix resin was prepared by mixing the Epocast PT100 (matrix) and Epoharden PT100S (curing agent) in a ratio of 2:1 by weight as per supplied guidelines by the manufacturer. Next, the composites were fabricated using a silicon mold, and alternate layers of matrix resin and CF strips were placed in that mold. After the last layer of the CF strip, the remaining resin was poured until it filled up the top of the mold. Figure 2 shows the schematic illustration of the composites with their dimensions. Finally, the samples were left at room temperature for 24 h to solidify and cure. Table 5 describes the composites produced in this process.

#### 2.2.4. Evaluation of Tensile Properties of Composites

Tensile strength and Young’s modulus of the composites were conducted using universal testing instruments (Shimadzu Autograph AGS–50kNX, Shidmadzu, Kyoto, Japan) following ASTM D638-14 [22] to evaluate the tensile properties. The cross-head speed was 0.5 mm/min; the sample’s test length was 40 mm (the measurement for test grip was excluded), while the width and thickness of the samples were 27 mm and 2 mm, respectively. Tensile strength, TS (N/mm^2^), was calculated using the following equation:

(1) $$\mathrm{TS}\;\left(\frac{N}{{\mathrm{mm}}^{2}}\right)=\frac{\mathrm{maximum}\;\mathrm{force}\;\mathrm{to}\;\mathrm{rupture}}{\mathrm{cross}\;\sec\mathrm{tion}}=\frac{F_{\max}}{A}\;$$

F_max_ (N) is the maximum force required to rupture the sample during the test, and A (mm^2^) is the sample’s cross-section calculated with the formula A=length ×width. Moreover, the stress—strain graph was plotted using the data obtained, and from the slope of the chart, the Young’s modulus (E) of the composite was determined.

#### 2.2.5. Fourier Transform Infrared (FTIR) Spectroscopy

In order to study the difference in the functional groups of the carbon fibers’ surfaces when the EPD factors were varied, FTIR analysis was done on a Shimadzu IRAffinity-1. Each sample was analyzed in the 4000–400 cm^−1^ regions with a resolution of 1 cm^−1^ [23,24].

#### 2.2.6. Scanning Electron Microscopy (SEM)

The surface morphology of the MWCNT–CF/epoxy composites was performed via scanning electron microscopy (SEM; Hitachi TM4000Plus Tabletop Microscope, Tokyo, Japan).

#### 2.2.7. Statistical and Graphical Analysis of Composite’s Tensile Properties

From the RSM in the Design Expert software, the fit statistics and mathematical model in terms of coded factors were used for the statistical analysis of the composites. Moreover, the three-dimensional (3D) response surface plot analysis (3D surface) and predicted versus actual graphs were obtained using graphical analysis. The chosen model was the cubic model, since there were three input factors in the experiment.

#### 2.2.8. Optimization of the Composite’s Tensile Properties

After the analysis, the data were optimized to obtain the optimal condition for the EPD process, maximizing the tensile strength and Young’s modulus of the composites. Both numerical and graphical optimization options were chosen in the Design Expert software for identifying the optimum values.

## 3. Results and Discussion

### 3.1. Characterization

#### 3.1.1. Functional Group Analysis

**Figure 3:**
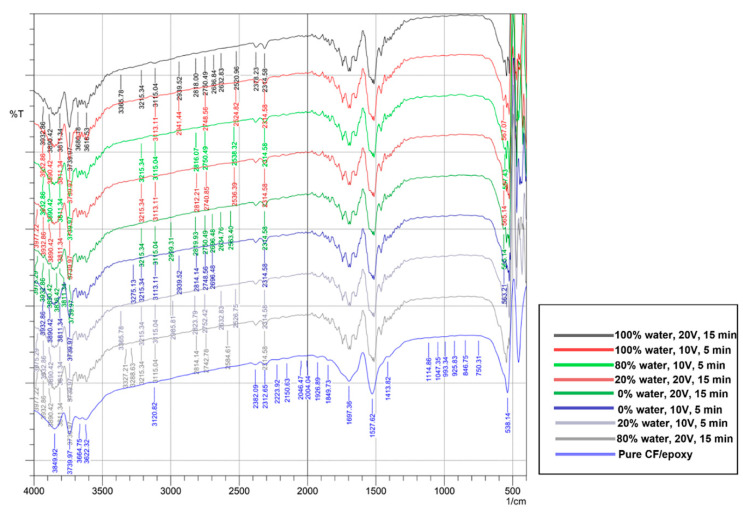
The FTIR analysis of the composites.

Figure 3 shows that the peaks obtained covered all regions from the fingerprint until the single bond region for the pure CF/epoxy composite. As for the composites reinforced with CNT, the identifiable peaks started from the triple bond region. For the pure CF/epoxy composite, the peaks from 900–670 cm^−1^ were ascribed to an aromatic C–H out-of-plane bend [25]. This was further verified by a study by Cecen et al. [26], where they ascribed an 828 cm^−1^ band to an aromatic ring bend out of the plane. In addition, apart from the pure CF/epoxy composite, the other samples exhibited peaks at around 2800 and continued till 2999 cm^−1^. It corresponded to C–H stretching, indicating the presence of methanol impurity [27]. Methanol was used in the study as the organic solvent for the EPD process, hence explaining the presence of methanol.

The presence of a peak around 2312 cm^−1^ in all the composites showed that carbon fiber and epoxy resin reacted strongly with one another. Thus, bonding occurred between the OH (hydroxyl) groups and carbon fiber and developed two reaction groups, namely, the epoxide and the hydroxyl group [26]. When MWCNTs were introduced in the CF/epoxy composite, a shift in peaks was observed from 3288 to 3313 cm^−1^, indicating strong O–H stretching. Therefore, the matrix and reinforcement had formed good interaction and dispersion [28].

#### 3.1.2. Surface Morphological Analysis

**Figure 4:**
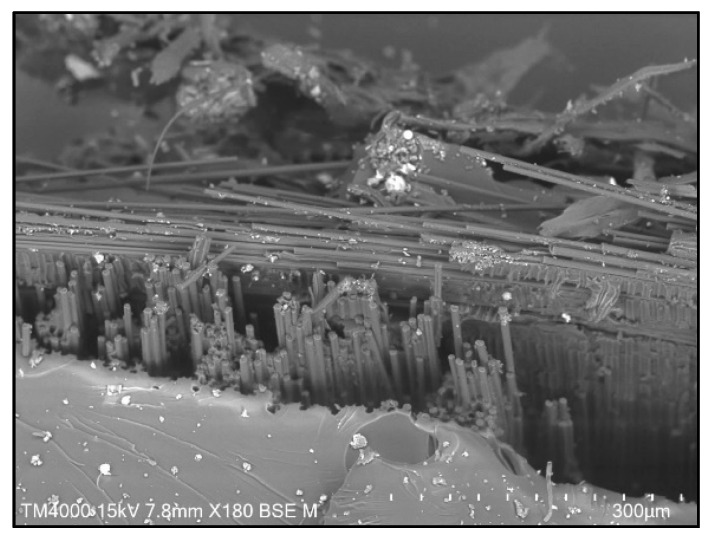
Surface morphology of pure CF/epoxy composite.

**Figure 5:**
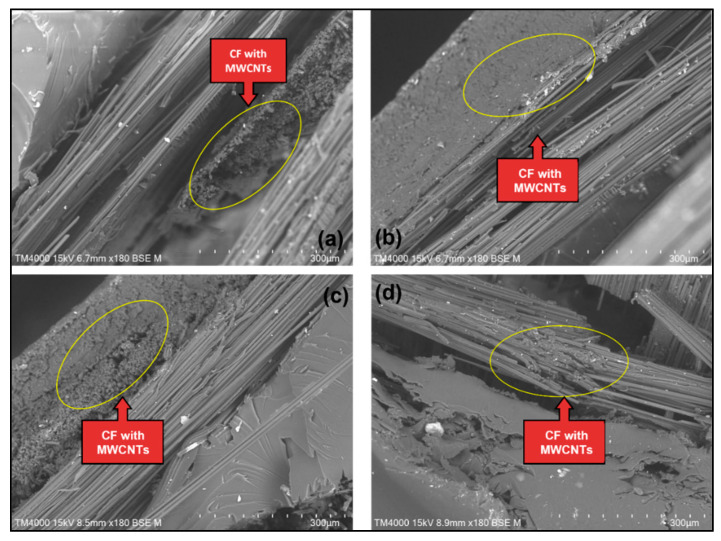
SEM images of CNT–CF/epoxy composites when deposition voltage is 20 V and deposition time is 15 min: (**a**) 0% water, (**b**) 20% water, (**c**) 80% water, (**d**) 100% water.

**Figure 6:**
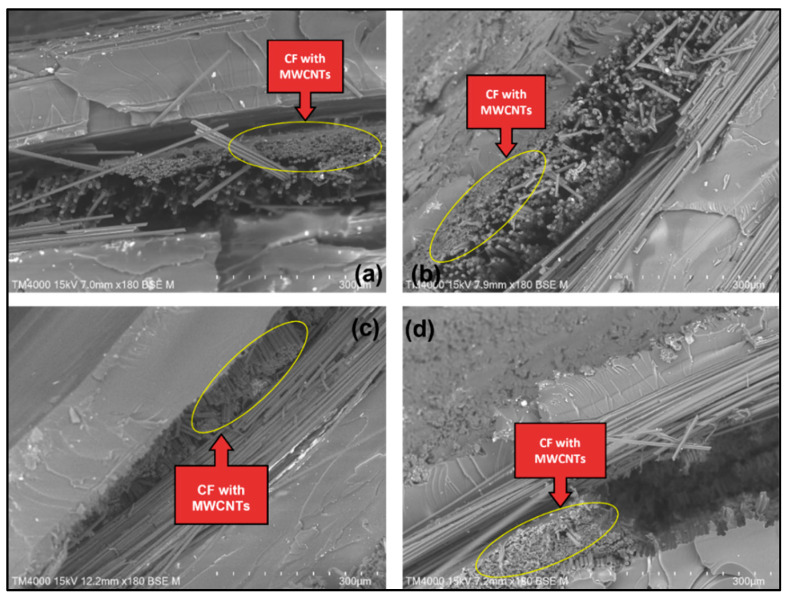
SEM images of CNT–CF/epoxy composites when deposition voltage is 10 V and deposition time is 5 min: (**a**) 0% water, (**b**) 20% water, (**c**) 80% water, (**d**) 100% water.

Figure 4 shows the surface of pure CF/epoxy composite, while Figure 5 and Figure 6 show the surface of MWCNT–CF/epoxy composites in different conditions. The figures show that the CFs remained attached to the epoxy matrix, indicating interfacial adhesion between the CF and epoxy. Though some voids can be seen in the images since no CFs were pulled out from the composites, the adhesion was deemed sufficient [29]. Additionally, the FTIR peak showed an interaction between CF and epoxy resin. The EPD process successfully deposited the MWCNTs, as the CFs were seen to be closely packed in the composite, shown in Figure 5 and Figure 6. However, the orientation of deposited MWCNTs was randomly distributed along the CF strips. The highest direct electric field of 20 V in this EPD process for all the composites is shown in Figure 5. It can be observed that the MWCNTs accumulation in the composites shown in Figure 5 was more than those in Figure 6. Excessive collection of similar charged electrons occurred when a high direct electric field was applied to the MWCNT–CF hybrid. As a result, the MWCNTs strongly repelled one another due to the exact electrical charges (high repulsion forces), forcing them to relocate into the most stable position at the CF strips [11]. In addition, incrementing the deposition time in this EPD process enhanced the MWCNTs in the vertical fiber, making it harder to pull out MWCNTs from the epoxy resin. Thus, the CF/epoxy interfacial adhesion was increased [30].

### 3.2. Tensile and Statistical Analysis of Composites’ Properties

#### 3.2.1. Tensile Properties of MWCNT–CF/Epoxy Composites

The evaluation of the hybrids’ applicability as polymeric matrix reinforcements was done by studying the mechanical properties of the composites. For example, the reinforcing effect of CF could be significantly improved when there were high compatibility and interaction between CF and matrix. Furthermore, with the addition of active nanomaterials such as MWCNTs, the interaction could be further enhanced [11]. The tensile properties of the pure CF/epoxy were individually tested, and the obtained values are listed in Table 6. Additionally, 36 specimens of the CNT–CF hybrid were prepared according to the experimental design. Table 7 and Table 8 show the trial conditions and results of the tensile testing of the fiber composites under various process factors.

Composites from design run 7, 10, 11, and 12 showed enhancement in the tensile strength compared to the pure CF/epoxy composite, with an increment of 30.87%, 52.59%, 39.34%, and 6.86%, correspondingly, as can be seen in Table 7 (0% and 100% water). As for the Young’s modulus, in comparison to the pure CF/epoxy, increments of 9.54%, 1.82%, 8.74%, and 8.77% was observed, respectively. From these composites, the composite from design run 10 (100% water, 20 V, 5 min) showed the highest improvement in terms of the tensile strength (52.59%) but relatively low improvement in Young’s modulus (1.82%). Moreover, as seen in Table 8 (20% and 80% water), compared to pure CF/epoxy, composites from design runs 6, 8, 9, 13, 16, and 18 showed improvements in their tensile strength with 1.39%, 2.59%, 24.49%, 38.26%, 37.14%, and 20.68%, accordingly. In addition, their Young’s modulus was enhanced by 6.44%, 4.41%, 4.99%, 0.36%, 4.33%, and 6.99%, respectively. From these composites, the composite from design run 13 (50% water, 15 V, 10 min) showed the highest improvement in terms of the tensile strength (38.26%) but little improvement in Young’s modulus (0.36%).

Generally, the matrix elongates as the tensile load increases, but the nanofillers (MWCNTs) resist the deformation. As a result, lower deformation occurs in the composite than in the neat polymer. Thus, higher tensile modulus and strength were obtained compared to the composite without any nanofiller, as the composites could sustain more loads [31]. The presence of MWCNTs helped the interface between layers to have good bonding with one another, as illustrated in the SEM presented in Figure 5 and Figure 6 [32]. The interfacial behavior of CF/epoxy was changed by introducing MWCNTs in the composite. The CF’s non-polar and inert surface properties caused weak interfacial adhesion between the woven CF and the epoxy matrix. Thus, the total CF/matrix interfacial area was decreased with the deposition of the MWCNTs on the woven CF surface, and the CNT/matrix interfacial area was increased instead. In addition, the stress transfer between CF and epoxy was improved due to the load-carrying capacity of CF with increased surface roughness. The twists and kinks created mechanical interlocks between MWCNTs and the epoxy matrix in the CNTs structure. As a result, the tensile strength of the MWCNT–CF/epoxy was improved, as MWCNTs had excellent mechanical properties [12]. However, since the improvement of the Young’s modulus was insufficient, this indicated that the agglomeration and orientation of MWCNTs formed on the CF surface were not sufficient to raise the interfacial interaction [11]. Therefore, it was implied that the overall composite’s Young’s modulus was not affected by adding nanostructure onto the epoxy system, though it increased the tensile strength of composites [33].

In addition, the composite from the design run 10 of 0% and 100% water (Table 7) showed that the applied voltage had a significant influence on increasing the tensile properties of the composite where the composite’s EPD was done at the highest voltage of 20 V but the lowest deposition time (5 min). Moreover, the composite from the design run 13 of 20% and 80% water (Table 8) demonstrated that for these reinforced composites, the applied time of the electric field during the deposition process had a direct influence on the tensile properties (10 min of the EPD process in 15 V applied voltage).

#### 3.2.2. Fit Statistics

Table 9 lists the fit statistics of both experimental designs, and the coefficient of variation, C.V., and prediction error sum of squares (PRESS) are shown in Table 10. From Table 9, it can be seen that the determination coefficients, R^2^, were relatively high, between 0.7 and 0.9. Generally, a good model fit has an R^2^ value of at least 0.8 [34]. When the R^2^ value is closer to unity (1), the model’s agreement with the experimental data is better, with a lesser difference between the calculated and measured values [35]. The obtained R^2^ values obtained were considered sufficient. Other than R^2^, adjusted R^2^, predicted R^2^, and prediction error sum of squares (PRESS) were also used to evaluate the model adequacy. A large R^2^ and low PRESS imply an excellent model [35]. A small value of PRESS is desired because it is used to determine the ability of the model to be used for future prediction [20]. However, only two PRESS values (30.25 and 1.455 × 10^5^) were considered fitting in this study.

Additionally, the difference between R^2^ and R^2^_adjusted_ must be evaluated to demonstrate the number of independent variables in the experiment. This study showed a significant difference between those two values, indicating that nominal terms had been included in the model [20]. The R^2^_adjusted_ was used to represent a rectified value for R^2^ after excluding unnecessary model terms. When the R^2^_adjusted_ was outstandingly smaller than R^2^, this implied a massive influx of non-significant terms present in the model [36]. Apart from that, the R^2^_predicted_ obtained in this study was primarily negative, which suggested that the overall mean should be a better response predictor [35].

Moreover, the coefficient of variation C.V. refers to the standard deviation to mean ratios. Therefore, the model is reproducible when the C.V. value is less than 10 [37]. For this study, only one of the C.V. values was less than 10, while the other three were slightly higher than 10. In addition, the signal-to-noise ratio (S/N ratio) was measured by adequate precision (AP). Therefore, when the AP value is greater than four, the model discrimination is considered satisfactory [38]. Moreover, the predicted values’ ranges at the design point to the average prediction error are compared using AP [34]. Therefore, the AP obtained from the experimental values could be considered adequate, as most values were around four, which is a good sign and implies that the model can be used to navigate the design space [37].

#### 3.2.3. Mathematical Model for Tensile Strength and Young’s Modulus (in Terms of Coded Factors)

Using the experimental results, the mathematical models for tensile strength and Young’s modulus for each experimental design were obtained (in terms of coded factors). The mathematical models were derived from the relationship between the input and output parameters, where the factors volume %, voltage, and time are represented as A, B, and C, respectively.

The following equations are the final mathematical models (in term of coded factors) for the experimental design 0% and 100% water:

**Tensile strength, TS:**

TS = 5.33 − 0.2134A − 1.55B − 0.9281C + 0.2492AB − 0.2050AC − 0.1025BC + 1.91A^2^ + 0.2180B^2^ − 1.57C^2^ − 0.3538ABC + 2.25A^2^B + 1.52A^2^C + 1.03AB^2^

**Young Modulus, E:**

E = 454.96 − 0.9508A − 32.800B + 8.112C + 0.075AB + 0.204AC + 0.394BC − 0.014A^2^ + 0.974B^2^ − 0.783C^2^ − 0.011ABC + 0.001A^2^B − 0.000039A^2^C − 0.0025AB^2^

Similarly, the following equations are the final mathematical models for tensile strength and Young’s modulus (in term of coded factors) for 20% and 80% water:

**Tensile strength, TS:**

TS = 6.31 + 1.48A − 0.0238B − 1.21C + 0.7073AB − 0.1431AC + 0.0657BC − 0.5027A^2^ + 1.22B^2^ − 2.05C^2^ + 0.7324ABC + 1.02A^2^B + 0.6604A^2^C − 1.18AB^2^

**Young Modulus, E:**

E = 249.39 − 11.18A − 11.46B − 39.59C + 4.52AB − 3.40AC + 3.92BC + 15.97A^2^ − 5.79B^2^ − 19.10C^2^ + 0.1325ABC + 8.29A^2^B + 20.26A^2^C + 16.49AB^2^

Additionally, the positive coefficient indicated a synergistic effect, while the negative coefficient demonstrated an antagonistic influence [39,40,41].

### 3.3. Graphical Analysis of Composites’ Mechanical Properties

#### 3.3.1. Response Surface Plots Analysis of Tensile Properties

**Figure 7:**
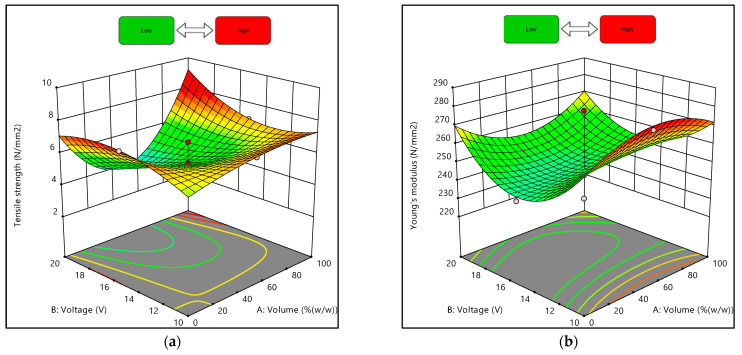
Three-dimensional response surface plots for experimental designs of 0% and 100% water: (**a**) Tensile strength, (**b**) Young’s modulus.

**Figure 8:**
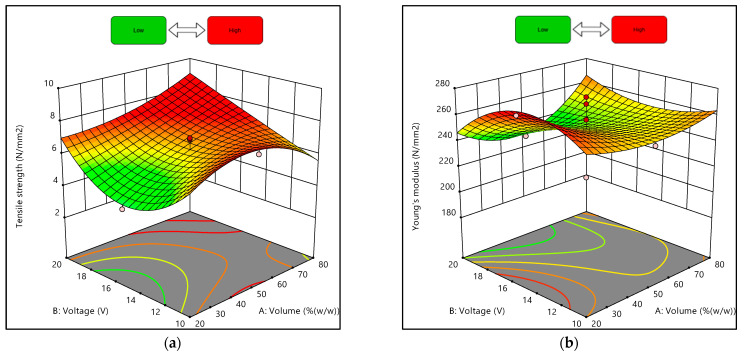
Three-dimensional response surface plots for experimental designs of 20% and 80% water: (**a**) Tensile strength, (**b**) Young’s modulus.

The effects of the interaction between the volume ratio of the suspension medium and the deposition voltage on the composites’ tensile strength and Young’s modulus under the conditions of 10 min of the EPD process are shown in Figure 7 and Figure 8. The response surface plot in Figure 7a shows that when the volume ratio of the suspension medium was increasing (approaching 100% water), the tensile strength also increased. Additionally, Figure 7b shows that when the voltages of the deposition process were at the maximum and minimum values, and the volume ratio increased, the Young’s modulus obtained was higher than the others.

Moreover, Figure 8a demonstrates that tensile strength increased as the volume ratio and voltage increased but slightly decreased when the volume ratio was approaching 80% water. In addition, Figure 8b illustrates that the Young’s modulus of the composites was comparatively high at lower voltage and higher volume ratios. Despite that, the obtained graphs were plotted with the deposition time being fixed as the actual factor at 10 min. As shown in Table 7 and Table 8, even when the volume ratios and deposition voltage were similar, the values of responses obtained differed depending on the deposition time.

#### 3.3.2. Prediction Versus Actual

**Figure 9:**
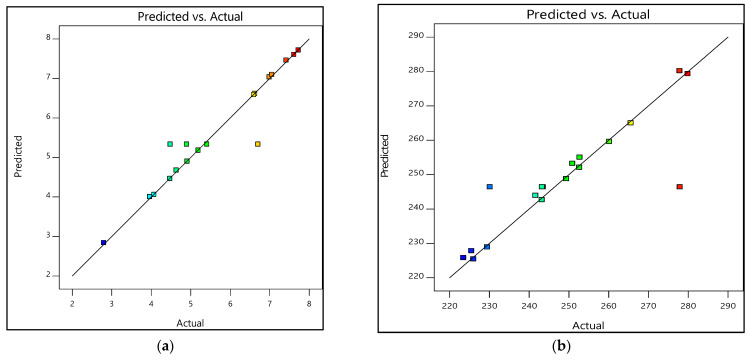
Prediction versus actual graphs for experimental designs of 0% and 100% water: (**a**) Tensile strength, (**b**) Young’s modulus.

**Figure 10:**
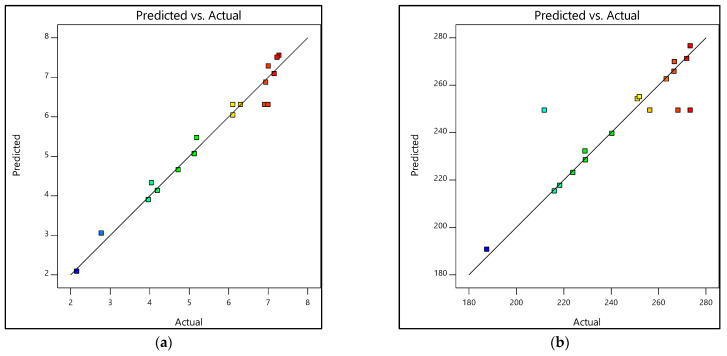
Prediction versus actual graphs for experimental designs of 20% and 80% water: (**a**) Tensile strength, (**b**) Young’s modulus.

The other response was the prediction versus actual. The responses of prediction versus actual graphs are demonstrated in Figure 9 and Figure 10. Furthermore, Table 11 and Table 12 show the experimental and predicted results of the output response, i.e., tensile strength and Young’s modulus. The residual values indicate the deviation between the actual and predicted values. If the experimental errors are random, the residuals are expected to follow a normal distribution [42]. As seen in Figure 9, the residuals formed a straight line, indicating a normal distribution of residuals of both tensile strength and Young’s modulus for the design of 0% and 100% water, whereas in Figure 10a, even though the tensile strength’s residuals formed slightly S-shaped curves, they could still be accepted. The curves were not significant enough to hold additional response transformation [42]. Additionally, most residual points were still close to the straight line. As for Figure 10b, the residuals were considered to follow the normal distribution except for a few points.

The percentage error was also calculated in Table 11 and Table 12. In Table 11, between the actual and predicted value for TS and E, the percentage error range of TS was approx. −18.94 to 20.45% and for E was approx. −7.06 to 11.35%. Additionally, for Table 12, the percentage error ranges of TS and E were as follows: TS approx. −9.9 to 9.9%, and E approx. −17.71 to 8.78%. Therefore, it can be deduced that experimental values were analogous to the predicted values as they were in close agreement [38]. Additionally, as the residuals matched the diagonal line, the created model was adequate [43].

### 3.4. Optimization of the EPD Process

**Figure 11:**
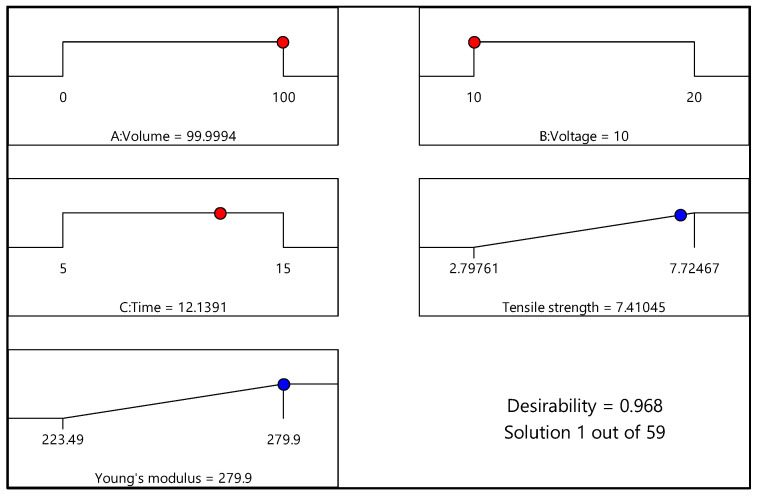
Ramp chart for statistically optimized factors and response for experimental design of 0% and 100% water.

**Figure 12:**
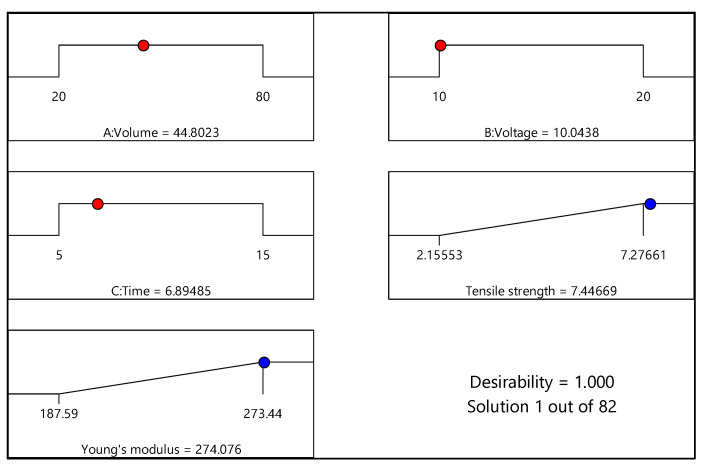
Ramp chart for statistically optimized factors and response for experimental design of 20% and 80% water.

Figure 11 and Figure 12 illustrate the numerical optimization results in terms of ramp graphs, while Table 13 and Table 14 list the constraints for the EPD process optimization. Numerical optimization uses a ramp graph to clarify the results. Generally, depending on the aim of the research, the design factors and responses are set to achieve the study’s objective [20]. For this study, the responses, namely, tensile strength and Young’s modulus, were set to the maximum option, and factors including volume ratio, voltage, and time were set in the range option. Based on Figure 11 (experimental design of 0% and 100% water), the optimum conditions for maximum responses were volume ratio (water as the basis) of 99.99%; deposition voltage of 10 V; and deposition time of 12.14 min. In addition, the desirability value was 0.968 for this response, close to 1. On the other hand, for the experimental design of 20% and 80% water, shown in Figure 12, the ideal conditions for the maximum responses were volume ratio (water as the basis) of 44.80%; deposition voltage of 10.04 V; and deposition time of 6.89 min. The desirability value for the experimental design was 1. Overall, both desirability values indicated that the formulation was desirable to fulfill the responses’ objectives, as the desirability value was close to one [44].

## 4. Conclusions

An EPD process to fabricate a CNT–CF hybrid was conducted using two DoE obtained from CCD and three parameters, i.e., volume ratios (water as the basis), deposition voltage, and deposition time. The laminated composites were manufactured using epoxy as the matrix and were varied with these design parameters. The effects of these three design parameters were studied on the tensile properties of the composites (tensile strength and Young’s modulus). The optimum conditions for the first DoE (0% and 100%) were achieved as a volume ratio of 99.99% water, deposition voltage of 10 V, and time of 12.14 min to obtain maximum tensile strength of 7.41 N/mm^2^ and Young’s modulus of 279.9 N/mm^2^. As for the second DoE (20% and 80%), tensile strength and Young’s modulus of 7.28 N/mm^2^ and 274.1 N/mm^2^, respectively, were achieved when the ideal conditions were volume ratio of 44.80% water, deposition voltage of 10.04 V, and 6.89 min. In conclusion, an ideal interaction between these three EPD parameters was necessary to achieve composites with good tensile properties.

## 5. Future Work

According to this study, various limitations might be seen throughout the research work. One of them is to alter the thickness of the sample specimen and learn how thickness affects it. Another kind of study that may be done is switching from water to deionized water to investigate the influence of altered medium on deposition. Other than that, increasing the test range below and beyond the designation range could be conducted to understand the behavior after the limitations, i.e., below 20% and after 80%.

## Figures and Tables

**Table 1:** Input factors used in the experiment for a volume ratio of 0 and 100% water.

Factor	Name	Units	Type	Minimum	Maximum	Coded Low (–)	Coded High (+)	Mean	Std. Dev.
A	Volume	% (*w*/*w*)	Numeric	0.0000	100.00	0.00	100.00	50.00	38.35
B	Voltage	V	Numeric	10.00	20.00	10.00	20.00	15.00	3.83
C	Time	min	Numeric	5.00	15.00	5.00	15.00	10.00	3.83

**Table 2:** Input factors used in the experiment for 20 and 80% water volume ratio.

Factor	Name	Units	Type	Minimum	Maximum	Coded Low (–)	Coded High (+)	Mean	Std. Dev.
A	Volume	% (*w*/*w*)	Numeric	20.00	80.00	20.00	80.00	50.00	23.01
B	Voltage	V	Numeric	10.00	20.00	10.00	20.00	15.00	3.83
C	Time	min	Numeric	5.00	15.00	5.00	15.00	10.00	3.83

**Table 3:** Experimental design for a volume ratio of 0%, 100% water.

Run	Factor 1 A: Volume %(*w*/*w*)	Factor 2 B: Voltage V	Factor 3 C: Time min
1	100	20	15
2	50	15	15
3	50	15	10
4	0	10	15
5	100	10	5
6	100	15	10
7	100	10	15
8	50	15	5
9	50	15	10
10	100	20	5
11	50	10	10
12	50	15	10
13	50	20	10
14	0	15	10
15	0	10	5
16	0	20	5
17	0	20	15
18	50	15	10

**Table 4:** Experimental design for a 20% and 80% water volume ratio.

Run	Factor 1 A: Volume %(*w*/*w*)	Factor 2 B: Voltage V	Factor 3 C: Time min
1	50	10	10
2	20	20	5
3	50	15	10
4	20	15	10
5	20	10	15
6	80	10	5
7	20	20	15
8	50	15	5
9	50	15	10
10	50	20	10
11	80	10	15
12	20	10	5
13	50	15	10
14	80	20	15
15	80	15	10
16	80	20	5
17	50	15	15
18	50	15	10

**Table 5:** Description of the samples.

Sample	Composite Description
Pure CF/epoxy composite	Epoxy laminated composite with 3 layers of woven CF
MWCNT–CF/epoxy composite	Epoxy laminated composite with 3 layers of woven CF reinforced with CNT

**Table 6:** Tensile strength and Young’s modulus of pure CF/epoxy composite.

Sample	Tensile Strength N/mm^2^	Young Modulus N/mm^2^
Pure CF/epoxy composite	5.062215	255.52

**Table 7:** Tensile strength and Young’s modulus of CNT–CF/epoxy composites with different factor values for 0% and 100% water.

Run	Factor 1 A: Volume %(*w*/*w*)	Factor 2 B: Voltage V	Factor 3 C: Time min	Response 1 Tensile Strength N/mm^2^	Response 2 Young Modulus N/mm^2^
1	100	20	15	7.61305	243.22
2	50	15	15	2.79761	225.49
3	50	15	10	6.70479	243.25
4	0	10	15	5.19014	229.48
5	100	10	5	4.91122	226.02
6	100	15	10	6.99095	250.91
7	100	10	15	6.62504	279.9
8	50	15	5	4.63372	223.49
9	50	15	10	4.48362	230.14
10	100	20	5	7.72467	260.16
11	50	10	10	7.05371	277.86
12	50	15	10	5.40961	277.92
13	50	20	10	3.96249	252.71
14	0	15	10	7.41783	241.61
15	0	10	5	4.07182	265.58
16	0	20	5	4.47299	249.36
17	0	20	15	6.59651	252.66
18	50	15	10	4.90024	243.5

**Table 8:** Tensile strength and Young’s Modulus of CNT–CF/epoxy composites with different factor values for 20% and 80% water.

Run	Factor 1 A: Volume %(*w*/*w*)	Factor 2 B: Voltage V	Factor 3 C: Time min	Response 1 Tensile Strength N/mm^2^	Response 2 Young Modulus N/mm^2^
1	50	10	10	7.27661	251.96
2	20	20	5	6.10913	240.39
3	50	15	10	6.91568	211.87
4	20	15	10	4.04905	273.44
5	20	10	15	4.72418	223.92
6	80	10	5	5.13248	271.98
7	20	20	15	3.96751	216.1
8	50	15	5	5.19331	266.78
9	50	15	10	6.30199	268.28
10	50	20	10	7.22907	229.04
11	80	10	15	2.15553	218.42
12	20	10	5	4.19924	263.34
13	50	15	10	6.99927	256.43
14	80	20	15	7.1577	229.22
15	80	15	10	7.00801	251.08
16	80	20	5	6.94222	266.59
17	50	15	15	2.77799	187.59
18	50	15	10	6.1089	273.39

**Table 9:** The fit statistics of the experimental responses.

Design of Experiment	Response	R^2^	Adjusted R^2^	Predicted R^2^	Adequate Precision
0% and 100% water	Tensile strength	0.9234	0.6743	0.1744	6.5963
	Young’s modulus	0.7687	0.0170	−13.3109	3.4278
20% and 80% water	Tensile strength	0.9688	0.8676	−24.2311	10.5406
	Young’s modulus	0.7825	0.0756	−11.9025	3.9312

**Table 10:** The C.V. and PRESS values of the responses.

Design of experiment	Response	C.V. %	PRESS
0% and 100% water	Tensile strength	14.85	30.25
	Young’s modulus	7.29	81,140.52
20% and 80% water	Tensile strength	10.56	1119.50
	Young’s modulus	10.13	1.455× 10^5^

**Table 11:** Experimental and predicted results of the output response for experimental design of 0% and 100% water.

Run	Volume Ratio	Voltage	Time	Tensile Strength (TS)				Young’s Modulus (E)			
				Actual	Pred	Residual	Error (%)	Actual	Pred	Residual	Error (%)
1	100	20	15	7.61	7.6	0.01	0.14	243.22	242.64	0.58	0.24
2	50	15	15	2.8	2.84	−0.04	−1.51	225.49	227.81	−2.32	−1.03
3	50	15	10	6.7	5.33	1.37	20.45	243.25	246.38	−3.13	−1.29
4	0	10	15	5.19	5.18	0.01	0.20	229.48	228.9	0.58	0.25
5	100	10	5	4.91	4.9	0.01	0.22	226.02	225.44	0.58	0.26
6	100	15	10	6.99	7.03	−0.04	−0.60	250.91	253.23	−2.32	–0.92
7	100	10	15	6.63	6.61	0.01	0.16	279.9	279.32	0.58	0.21
8	50	15	5	4.63	4.68	−0.04	−0.91	223.49	225.81	−2.32	−1.04
9	50	15	10	4.48	5.33	−0.85	−18.94	230.14	246.38	−16.24	−7.06
10	100	20	5	7.72	7.71	0.01	0.14	260.16	259.58	0.58	0.22
11	50	10	10	7.05	7.1	−0.04	−0.60	277.86	280.18	−2.32	−0.83
12	50	15	10	5.41	5.33	0.08	1.43	277.92	246.38	31.54	11.35
13	50	20	10	3.96	4	−0.04	−1.07	252.71	255.03	−2.32	−0.92
14	0	15	10	7.42	7.46	−0.04	−0.57	241.61	243.93	−2.32	−0.96
15	0	10	5	4.07	4.06	0.01	0.26	265.58	265	0.58	0.22
16	0	20	5	4.47	4.46	0.01	0.24	249.36	248.78	0.58	0.23
17	0	20	15	6.6	6.59	0.01	0.16	252.66	252.08	0.58	0.23
18	50	15	10	4.9	5.33	−0.43	−8.82	243.5	246.38	−2.88	−1.18

**Table 12:** Experimental and predicted results of the output response for 20% and 80% experimental water design.

Run	Volume Ratio	Voltage	Time	Tensile Strength (TS)				Young’s Modulus (E)			
				Actual	Pred	Residual	Error (%)	Actual	Pred	Residual	Error (%)
1	50	10	10	7.28	7.55	−0.28	−3.78	251.96	255.06	−3.10	−1.23
2	20	20	5	6.11	6.04	0.07	1.13	240.39	239.61	0.78	0.32
3	50	15	10	6.92	6.31	0.61	8.80	211.87	249.39	−37.52	−17.71
4	20	15	10	4.05	4.32	−0.28	−6.79	273.44	276.54	−3.10	−1.13
5	20	10	15	4.72	4.66	0.07	1.46	223.92	223.14	0.78	0.35
6	80	10	5	5.13	5.06	0.07	1.34	271.98	271.2	0.78	0.29
7	20	20	15	3.97	3.9	0.07	1.73	216.1	215.32	0.78	0.36
8	50	15	5	5.19	5.47	−0.28	−5.30	266.78	269.88	−3.10	−1.16
9	50	15	10	6.3	6.31	0.00	−0.07	268.28	249.39	18.89	7.04
10	50	20	10	7.23	7.5	−0.28	−3.80	229.04	232.14	−3.10	−1.35
11	80	10	15	2.16	2.09	0.07	3.19	218.42	217.64	0.78	0.36
12	20	10	5	4.2	4.13	0.07	1.64	263.34	262.56	0.78	0.29
13	50	15	10	7	6.31	0.69	9.90	256.43	249.39	7.04	2.75
14	80	20	15	7.16	7.09	0.07	0.96	229.22	228.44	0.78	0.34
15	80	15	10	7.01	7.28	–0.28	−3.92	251.08	254.18	−3.10	−1.23
16	80	20	5	6.94	6.87	0.07	0.99	266.59	265.81	0.78	0.29
17	50	15	15	2.78	3.05	–0.28	−9.90	187.59	190.69	−3.10	−1.65
18	50	15	10	6.11	6.31	–0.20	−3.23	273.39	249.39	24.00	8.78

**Table 13:** Constraints for optimization for experimental design of 0% and 100% water.

Constraints	Conditions	Lower Limit	Upper Limit	Solution
Volume ratio, % (*w*/*w*)	In range	0	100	99.99
Voltage (V)	In range	10	20	10
Time (min)	In range	5	15	12.14
Tensile strength	Maximize	-	-	7.41
Young’s modulus	Maximize	-	-	279.9

**Table 14:** Constraints for optimization for 20% and 80% experimental water design.

Constraints	Conditions	Lower limit	Upper Limit	Solution
Volume ratio, % (*w*/*w*)	In range	20	80	44.80
Voltage (V)	In range	10	20	10.04
Time (min)	In range	5	15	6.89
Tensile strength	Maximize	-	-	7.28
Young’s modulus	Maximize	-	-	274.1
